# Association of serum 25-Hydroxyvitamin D with Vitamin D intervention and outdoor activity among children in North China: an observational study

**DOI:** 10.1186/s12887-020-02435-9

**Published:** 2020-12-02

**Authors:** Xuguang Zhang, Yanping Chen, Shanshan Jin, Xinxin Bi, Dongkai Chen, Dongmei Zhang, Li Liu, Hong Jing, Lixin Na

**Affiliations:** 1 Department of Child Healthcare, Harbin Children’s Hospital, Harbin, 150010 China; 2 Collaborative Innovation Center for Biomedicine, Medical Technology College, Shanghai University of Medicine & Health Sciences, Shanghai, 201318 China; 3 Health Supervision Institute of Harbin Municipal Health Bureau, Harbin, 150010 China

**Keywords:** Vitamin D, Therapeutic intervention, Supplementation intervention, Outdoor activity, Children, North China

## Abstract

**Background:**

Living at high latitudes is one of the risk factors for vitamin D deficiency in children. However, evidence on vitamin D improvement for this pediatric population to date is limited. This study aims at evaluating the association of different vitamin D intervention methods and outdoor activity on the vitamin D status of children in North China.

**Methods:**

In this observational study, a total of 55,925 children aged 1 month to 18 years old were recruited from pediatric outpatient departments from July 2016 to June 2017. Data on demographics, anthropometric measurements, vitamin D intervention (either prescribed by physicians or given by parents) and outdoor activity were recorded. The serum levels of 25-hydroxycholecalciferol (25(OH)D) were determined by high performance liquid chromatography tandem–mass spectrometry. Logistic regression analysis was performed to assess the association of vitamin D intervention or outdoor activity with blood vitamin D status, adjusted for age, gender, BMI for age, and seasons.

**Results:**

The overall rate of hypovitaminosis D was 65.60%. Of the children’s outdoor activity, 35.63, 31.95, and 32.42% were below 30 min/d, 30–60 min/d and over 60 min/d, respectively. Furthermore, the proportion of therapeutic intervention, supplementation intervention and no vitamin D intervention among the children was 16.48, 32.87, and 50.65%, respectively. After adjusted for confounding factors, vitamin D intervention was associated with a lower risk of hypovitaminosis D, with OR (95% CI) of 0.191 (0.180, 0.202) in children with therapeutic doses and 0.423 (0.404, 0.443) in those with supplementation doses, compared with children without vitamin D intervention. In addition, longer outdoor time was associated with a lower risk of hypovitaminosis D [0.479 (0.456, 0.504) for 60 min/d, 0.737 (0.701, 0.776) for 30–60 min/d], independent of vitamin D intervention.

**Conclusions:**

High prevalence of vitamin D deficiency was found in children living at high latitudes. Vitamin D intervention and outdoor activity are all negatively associated with children’s vitamin D deficiency. Routine vitamin D intervention combined with increased outdoor time might be an effective approach to prevent hypovitaminosis D among children, especially those at school, living at high latitudes.

**Supplementary Information:**

The online version contains supplementary material available at 10.1186/s12887-020-02435-9.

## Background

Vitamin D deficiency is a disease mainly induces disorders of calcium and phosphorus metabolism, which causes defective chondrocyte differentiation, mineralization in growth plate and defects in osteoid mineralization among children [1]. Vitamin D deficiency is also related to functions of muscles and immune organs, and hematopoiesis, which are associated with multiple extra-skeletal disorders, such as myopathy, anemia, and recurrent respiratory tract infections in children [2–4].

Vitamin D deficiency or insufficiency are still of global public health concerns, particularly in developing countries [5]. Approximately 30% of children suffer from hypovitaminosis D worldwide [6]. Academic societies of China and other regions have released guidelines to emphasize the role of outdoor time and vitamin D intervention (routine vitamin D supplementation in the first year of life and for those aged 1–18 years with risk factors for vitamin D deficiency) in physical health of children and adolescents [7, 8]. Several studies have demonstrated that vitamin D intervention significantly increased blood 25-hydroxyvitamin D (25(OH)D) level in children. A randomized, double-blinded study reported that vitamin D treatment in 600 IU/d for 6 weeks resulted in an increase in serum 25(OH) D in asthmatic children compared with placebo treatment (76.3 vs 48.2 nmol/l) [9]. In a prospective observational study, 55 children with inflammatory bowel diseases were prescribed vitamin D 2000 IU/d for 2 to 3 months, and their serum 25(OH) D were increased from 58 nmol/L at the baseline to 85 nmol/L. [10] Uysalol et al. found that serum 25(OH) D levels in asthmatic children with inadequate sun exposure and vitamin D intervention were significantly lower than that of the healthy controls (16.6 vs 28.2 ng/mL) in Turkey [11]. Therefore, most present studies focused on the impact of vitamin D intervention and outdoor time on pediatric patients. Yet the association of vitamin D intervention and sun exposure on healthy children are not well documented given that few guidelines support serum 25(OH) D test for children [12]. However, previous studies suggested that circulating vitamin D level should be measured even in healthy children with a risk of vitamin D deficiency mainly due to inadequate sun exposure [13, 14], in order to early prevent vitamin D deficiency-related diseases.

Constant exposure of many parents to information on benefits of vitamin D supplementation during pregnancy and throughout childhood may contribute to voluntary daily intake of vitamin D in children [15, 16]. Supplementation intervention of vitamin D helps prevent vitamin D deficiency in children, although few studies have been reported [17]. With the spread of child health education, children are more likely to have regular clinic visits due to early recognition of nutritional rickets-related clinical symptoms by their parents, e.g. frontal bossing, rachitic rosary, and leg deformity. 25(OH) D test is routinely performed for children in North China as part of physical examination or assessment of suspected nutritional rickets. Once the diagnosis is confirmed, individualized intervention is provided by physicians/doctors to maintain or improve vitamin D status of their pediatric patients. Nevertheless, little research has been conducted on efficacy and feasibility of therapeutic intervention to date. In our study, data of 55,925 children from our clinic were analyzed to investigate their vitamin D nutriture and possible causes, of which vitamin D intervention and outdoor time were of particular interest, to explore the necessity of regular monitoring of 25(OH) D status and intervention in children living at high latitudes.

## Methods

### Study design, data source, and patient selection

Heilongjiang is a province comprising 12 cities including Harbin, located in North China at latitude 43°3′ - 53°3′ N and known as the coldest region in China, with an average annual sunshine of 1874 to 2761 h in 2016–2017, according to Heilongjiang Bureau of Statistics. The Sixth National Population Census of China indicated that the resident population in Heilongjiang was 38.31 million, in which 4.57 million (11.94%) were aged 0–14 years old.

In China, child health is a branch of pediatric medicine mainly dealing with physical examination and health assessment on infants and young children including growth and the behavioral development assessment, feeding guidance, assessment of nutritional status, micronutrient testing, as well as diagnosis and treatment of rickets. In this cross-sectional study, children aged 0–18 years old were recruited who visited child health clinic in Harbin Children’s Hospital between July 2016 and June 2017. All guardians completed questionnaires involving questions of their children’s name, date of birth, date of visit, ethnic, and vitamin D intervention.

Therapeutic intervention is defined as vitamin D prescription in children who had physical examination or clinic visits in the past 3–6 months. Prophylactic and therapeutic dosage was recommended for children with a normal level and in vitamin D deficiency, respectively according to the consensus of the Chinese Society of Osteoporosis and Bone Mineral Research [18], respectively (most of them had oral vitamin D3 intake of 800–2400 IU/d). Supplementation intervention is defined as daily vitamin D intake in children asked by their parents in last 3–6 months (most of them had oral vitamin D3 intake of 400–800 IU/d), without being monitored for their serum 25(OH) D or a prescription from doctors. No vitamin D intervention was defined as taking no vitamin D supplements in the past 3–6 months. Children were considered being supplemented with vitamin D if they were 1) aged over 6 months and given either therapeutic or supplementation vitamin D intervention for more than 3 months and 2) aged less than 6 months and received vitamin D intervention in either aforementioned way for > 1 month. Children with the following conditions were excluded from our study: 1) using vitamin D metabolites and their analogs, e.g., alfacalcidol or calcitriol, for their routine supplementation. 2) suffering from hypophosphatemic rickets, organ dysfunction, congenital disorders, inherited metabolic diseases, acute infectious diseases and chronic inflammatory disease. 3) for those over 6 months old, either aforementioned intervention suspended for > 20 d in the past 3 months and 4) for children under 6 months, either aforementioned intervention suspended for > 10 d in the past 1 month. Detailed information on the nature of this study was provided to the parents or guardians of participants before consent was written.

### Outdoor activity

The questionnaire about outdoor activity time included the average frequency of outdoor activity per week in the past 3 months (1 to 7 days), duration per day (less than 30 min/ day, 30 ~ 60 min/ day and more than 60 min/ day) and activity window (before 8 a.m., 8 a.m. to 4 p.m. and post 4 p.m.). Outdoor activity from 8 a.m. to 4 p.m. was considered as being associated with vitamin status and investigated. Pre-investigation of 50 randomly-selected parents was conducted and repeated 1 week afterwards, resulting in the reliability coefficient *r* = 0.912.

### Anthropometric measurements

Trained nurses measured twice the height and weight of the enrolled children within 0.1 cm and 0.1 kg respectively, and average the measurements for the final value. An anthropometric calculator obtained from World Health Organization (http://www.who.int/en/) was used to determine body mass index (BMI) for age. BMI for age < 90 percentile was considered normal, 90–97 percentile overweight and > 97 percentile obese.

### Circulating 25(OH) D level

Blood samples (3 ml) were taken by antecubital venipuncture and then stored in a cool box for < 2 h. After centrifugation at room temperature for 10 min at 1200×*g*, the upper serum pipetted and stored at 4 °C for < 24 h. The samples were kept at room temperature for 30 min before analysis. The serum 25(OH) D levels were determined by high performance liquid chromatography tandem–mass spectrometry (HPLC–MS/MS, API 3200; AB SCIEX, American). The lower limits of 25-(OH)D2 and 25-(OH)D3 for detection were both 1.6 ng/mL. The test sensitivity was assessed with the inter-batch coefficient of variation (CV) of 6.59% and between batches CV of 6.98%.

### Diagnostic criteria of serum Vitamin D status

The consensus of the Chinese Society of Osteoporosis and Bone Mineral Research, Chinese Medical Association indicated that circulating 25(OH) D < 10 ng/mL was considered severely deficient, 10–20 ng/mL deficient, 20–29 ng/mL insufficient, and ≥ 30 ng/mL sufficient [18]. High levels of circulating 25(OH) D may have benefits for children’s extra-skeletal organs [19]. Thus, the cut-off points for defining low status of vitamin D is 30 ng/mL, which refers to hypovitaminosis D in this study [14].

### Statistical analysis

The serum level of 25(OH) D was expressed as mean ± SD. Comparison of the 25(OH) D levels between different groups was determined by ANOVA. The prevalence of vitamin D status was expressed as *n* (%). The statistical difference of the prevalence of vitamin D status between different groups was determined by chi-square test. Logistic regression analysis was performed to evaluate the association of risk factors with blood vitamin D status. Furthermore, the associations of hypovitaminosis D with vitamin D intervention and outdoor time was examined after adjustment for age, gender, BMI for age, and season. Data were expressed as odds ratio (OR) and 95% confidence interval (CI). All analyses were performed using SPSS 21.0. A *p* value < 0.05 was considered statistically significantly.

## Results

### Participant characteristics

A total of 55,925 participants from the 12 cities of Heilongjiang were included in this study, in which 30,683 (54.86%) were male. Table 1 presented the basic characteristics of the participants. The average serum level of 25(OH) D for all participants was 26.13 ± 12.30 ng/mL. The median age of participants was 3.10 years old (interquartile range, 1.10–6.50 years old). The highest level of serum vitamin D was found in the group younger than 1 year old. As the age increased, the serum vitamin D level was dropped, which remained 16.24 ± 8.30 ng/mL in children aged from 12 to younger than 18 years old. The serum vitamin D levels in normal-weight, overweight and obese children were 26.58 ± 12.26 ng/mL, 21.84 ± 12.43 ng/mL and 20.07 ± 9.90 ng/mL (*P <* 0.001), respectively, indicating the deterioration in vitamin D status with increasing body weight in children. Children had the lowest level of vitamin D (25.37 ± 11.95 ng/mL, *P* < 0.001) in winter than in other seasons of the year. There was no significant difference of serum vitamin D between the Han children and the minority peers (26.12 ± 12.29 vs 26.91 ± 12.67 ng/mL, *P* = 0.050). Of the children’s outdoor time, 35.63, 31.95, and 32.42% were below 30 min/d, 30–60 min/d and over 60 min/d, respectively. Children with more than 60 min/d outdoor activity had the highest level of serum vitamin D (29.60 ± 12.11 ng/mL, *P <* 0.001). Furthermore, 50.65% of the subjects were not given vitamin D intervention (20.60 ± 8.65 ng/mL), 32.87% received vitamin D supplementation (29.80 ± 9.49 ng/mL) and 16.48% were provided therapeutic doses of vitamin D (35.80 ± 17.14 ng/mL, *P* < 0.001).

**Table 1:** 25(OH) D concentration in children aged 0–18 years old

	N (%)	25(OH) D (ng/mL)	*P* value
Total	55,925	26.13 ± 12.30	
Gender			< 0.001
Boys	30,683 (54.86)	26.42 ± 12.07	
Girls	25,241 (45.13)	25.78 ± 12.56	
Age (year)			< 0.001
0 < age < 1	11,953 (21.37)	33.25 ± 13.64	
1 ≤ age < 3	14,901 (26.64)	30.71 ± 11.44	
3 ≤ age < 6	13,124 (23.47)	23.74 ± 9.37	
6 ≤ age < 12	12,416 (22.20)	19.12 ± 8.70	
12 ≤ age < 18	3531 (6.31)	16.24 ± 8.30	
Season			< 0.001
Spring	15,795 (28.24)	25.98 ± 11.77	
Summer	17,421 (31.15)	26.58 ± 13.07	
Autumn	10,205 (18.25)	26.51 ± 12.08	
Winter	12,504 (22.36)	25.37 ± 11.95	
Ethnic			0.050
Han	54,967 (98.29)	26.12 ± 12.29	
Ethnic minorities in China	958 (1.71)	26.91 ± 12.67	
Outdoor time			< 0.001
< 30 min/d	19,929 (35.63)	21.61 ± 11.81	
30–60 min/d	17,867 (31.95)	27.65 ± 11.47	
> 60 min/d	18,129 (32.42)	29.60 ± 12.11	
BMI for age			< 0.001
Normal	51,294 (91.72)	26.58 ± 12.26	
Overweight	2851 (5.10)	21.84 ± 12.43	
Obesity	1780 (3.18)	20.07 ± 9.90	
Intervention			< 0.001
None	28,328 (50.65)	20.60 ± 8.65	
Supplementation intervention	18,383 (32.87)	29.80 ± 9.49	
Therapeutic intervention	9214 (16.48)	35.80 ± 17.14	

NOTE: *P* < 0.05 shows a significant difference between the groups

### Characteristics of children with different Vitamin D statuses

The overall rate of hypovitaminosis D was 65.60%. 6.57, 25.51, and 33.52% of the children included were found with severe deficiency, deficiency and insufficiency, respectively. Moreover, 18.11% children with severe deficiency were overweight or obese, compared with 11.34 and 8.17% in those with deficiency and insufficiency, respectively. However, the rate of overweight and obesity decreased to 4.21% in children with vitamin D sufficiency (*P <* 0.001). Children who have outdoor time of 30–60 min/d and over 60 min/d showed a percentage of 34.31 and 39.97% in the vitamin D sufficiency group, whereas it dropped to 25.72% in the < 30 min/d group (*P <* 0.001). In children with normal vitamin D status, 46.70 and 27.12% had supplementation and therapeutic doses of vitamin D, respectively; in contrast, 26.18% received no intervention, accounting for 90.31% patients with severe vitamin D deficiency (Table 2).

**Table 2:** Characteristics of children with varying serum levels of 25(OH)D

	Severe deficient	Deficient	Insufficient	Sufficient	*P* value
Vitamin D (ng/mL,^−^x ± s)	7.45 ± 2.19	15.35 ± 2.87	25.04 ± 2.81	38.74 ± 10.57	< 0.001
Percentage (%)	6.57	25.51	33.52	34.40	
Male (%)	51.20	53.07	55.42	56.34	< 0.001
Age (years,^−^x ± s)	7.54 ± 4.99	6.65 ± 3.94	4.18 ± 3.38	2.09 ± 2.39	< 0.001
Ethnic (Han, %)	98.31	98.49	98.28	98.14	0.063
Overweight and obesity (%)	18.11	11.34	8.17	4.21	< 0.001
Season (%)					< 0.001
Spring	30.50	29.82	31.12	32.29	
Summer	26.53	29.33	28.19	27.83	
Autumn	16.31	17.35	18.69	18.85	
Winter	26.66	23.50	22.00	21.03	
Outdoor activity time (%)					< 0.001
< 30 min/d	58.69	56.23	25.63	25.72	
30–60 min/d	23.56	22.14	38.63	34.31	
> 60 min/d	17.75	21.63	35.74	39.97	
Intervention methods (%)					< 0.001
No intervention	90.31	74.10	50.16	26.18	
Supplementation intervention	7.27	17.94	35.06	46.70	
Therapeutic intervention	2.43	7.96	14.79	27.12	

^−^x ± s and percentage (%) were used to describe the continuous and categorical variables, respectively; *P* < 0.05 was regarded as statistically significant

### Association of Risk Factors with Vitamin D status

With the logistic regression model, we analyzed the risk factors including gender, age, ethnics, BMI for age, season, outdoor time and vitamin D intervention for prediction of hypovitaminosis D in all the participants (Table 3). The results showed that among the influential factors, female (OR = 1.155, *P* < 0.001, relative to males), older ages (1 ≤ age < 3, OR = 1.534, *P* < 0.001; 3 ≤ age < 6, OR = 5.779, *P* < 0.001; 6 ≤ age < 12, OR = 12.685, *P* < 0.001; 12 ≤ age < 18, OR = 15.932, *P* < 0.001; relative to those younger than 1 years old), overweight and obesity (OR = 1.219, *P* < 0.001, relative to normal) and winter (OR = 1.189, *P* < 0.001, relative to summer) were associated with an increased risk of hypovitaminosis D. In contrast, increased outdoor activity time (30–60 min/d, OR = 0.756, *P* < 0.001; > 60 min/d, OR = 0.482, *P* < 0.001; relative to < 30 min/d) and vitamin D intervention (supplementation intervention, OR = 0.416, *P* < 0.001; therapeutic intervention, OR = 0.183, *P* < 0.001; relative to no intervention) were associated with an decreased risk of hypovitaminosis D.

**Table 3:** Regression analysis of risk factors for Hypovitaminosis D

	OR (95% CI, *n* = 55,925)	*P* Value
Gender		
Male	1	
Female	1.155 (1.108, 1.205)	< 0.001
Age (years)		
0 < age < 1	1	
1 ≤ age < 3	1.534 (1.457, 1.615)	< 0.001
3 ≤ age < 6	5.779 (5.437, 6.142)	< 0.001
6 ≤ age < 12	12.685 (11.740, 13.707)	< 0.001
12 ≤ age < 18	15.932 (13.738, 18.476)	< 0.001
Ethnic group		
Han	1	
Other	1.709 (0.923, 1.260)	0.341
BMI for age		
Normal	1	
Overweight and obesity	1.219 (1.046, 1.421)	< 0.001
Season group		
Summer	1	
Spring	1.079 (1.017, 1.145)	0.141
Autumn	0.957 (0.903, 1.015)	0.012
Winter	1.189 (1.113, 1.270)	< 0.001
Outdoor time		
< 30 min/d	1	
30–60 min/d	0.756 (0.718, 0.797)	< 0.001
> 60 min/d	0.482 (0.458, 0.508)	< 0.001
Intervention		
None	1	
Supplementation intervention	0.416 (0.396, 0.437)	< 0.001
Therapeutic intervention	0.183 (0.173, 0.194)	< 0.001

### Association of Intervention Methods with Vitamin D status

After adjusting for age, sex, BMI for age, season, and outdoor activity time, an inverse association of vitamin D intervention with hypovitaminosis D was observed. The OR (95% CI) of hypovitaminosis D was 0.423 (0.404, 0.443) in children with supplementation doses and 0.191 (0.180, 0.202) in children with therapeutic doses compared with children with no supplements (Table 4). After stratification by age group, the negative association of vitamin D interventions (including supplementation and therapeutic doses) with hypovitaminosis D existed in each age group (Supplement Table 1).

**Table 4:** Association of vitamin D intervention with hypovitaminosis D

Intervention	Model 1	Model 2	Model 3
No intervention	1	1	1
Supplementation intervention	0.435 (0.415, 0.455)	0.435 (0.415, 0.455)	0.423 (0.404, 0.443)
Therapeutic intervention	0.202 (0.191, 0.214)	0.202 (0.191, 0.214)	0.191 (0.180, 0.202)

Associations were examined with multivariable logistic regression. Model 1: adjusted for age and sex. Model 2: adjusted for BMI for age on the basis of model 1. Model 3: adjusted for season and outdoor time on the basis of model 2

### Association of the Intervention Methods with Vitamin D status in children with varying outdoor time

We conducted logistic regression to further eliminate the influence of outdoor activity time on the relationship between intervention methods and serum 25(OH) D level. Both therapeutic and supplementation intervention reduced the risk of hypovitaminosis D regardless of outdoor time compared to those with no vitamin D intervention. In children whose outdoor time was > 60 min/d, the OR was 0.196 (0.178, 0.216) in the former way, whereas 0.508 (0.470, 0.550) in the latter way, compared with those who were not given intervention (Table 5).

**Table 5:** Logistic regression analysis of the association of vitamin D intervention methods with hypovitaminosis D in children with varying outdoor time

Intervention methods	Model 1	Model 2	Model 3
> 60 min/d			
No intervention	1	1	1
Supplementation intervention	0.508 (0.470, 0.549)	0.508 (0.469, 0.549)	0.508 (0.470, 0.550)
Therapeutic intervention	0.196 (0.177, 0.216)	0.196 (0.177, 0.216)	0.196 (0.178, 0.216)
30–60 min/d			
No intervention	1	1	1
Supplementation intervention	0.362 (0.332, 0.394)	0.362 (0.332, 0.394)	0.361 (0.332, 0.394)
Therapeutic intervention	0.183 (0.164, 0.205)	0.184 (0.164, 0.205)	0.183 (0.164, 0.205)
< 30 min/d			
No intervention	1	1	1
Supplementation intervention	0.376 (0.345, 0.411)	0.377 (0.346, 0.411)	0.377 (0.346, 0.411)
Therapeutic intervention	0.176 (0.160, 0.194)	0.174 (0.159, 0.192)	0.174 (0.159, 0.192)

Associations were examined using multivariable logistic regression. Model 1: adjusted for age and sex. Model 2: adjusted for BMI for age on the basis of model 1. Model 3: adjusted for season on the basis of model 2

### Association of Outdoor Time with Vitamin D status

We found another inverse association of prolonged outdoor time with hypovitaminosis D after age, sex, BMI for age, season, and intervention methods were adjusted. The OR (95% CI) of hypovitaminosis D was 0.737 (0.701, 0.776) in children with outdoor time of 30–60 min/d and 0.479 (0.456, 0.504) in those with 60 min/d compared to those with less than 30 min (Table 6). Besides, after age stratification, prolonged outdoor time was negatively associated with hypovitaminosis D in all age groups (Supplement Table 2).

**Table 6:** Logistic regression analysis of the association of outdoor time with hypovitaminosis D

Outdoor time (min/d)	Model 1	Model 2	Model 3
< 30	1	1	1
30–60	0.696 (0.663, 0.731)	0.697 (0.663, 0.732)	0.737 (0.701, 0.776)
> 60	0.533 (0.508, 0.560)	0.534 (0.508, 0.560)	0.479 (0.456, 0.504)

Associations were examined using multivariable logistic regression. Model 1: adjusted for age and sex. Model 2: adjusted for BMI for age on the basis of model 1. Model 3: adjusted for season and intervention methods on the basis of model 2

## Discussion

This observational study was the first to assess circulating 25(OH) D in over 50,000 children by HPLC–MS/MS, the current gold standard in vitamin D level assessment [20]. Our data showed that (1) hypovitaminosis D was prevalent (65.60%) among children in Heilongjiang; (2) older ages, winter and overweight and obesity were risk factors for pediatric hypovitaminosis D. (3) supplementation and therapeutic vitamin D intervention were inversely associated with the rates of pediatric hypovitaminosis D; (4) increased outdoor activity time was also linked with a lower risk of hypovitaminosis D, and (5) vitamin D intervention combined with outdoor time over 60 min/d may be a better way in preventing hypovitaminosis D among children in North China.

In this study, there was no significant difference of serum vitamin D between Han children and the minority group. This corresponds with a cross-sectional study involving 14,473 children aged 6 ~ 17 years old from 31 provinces in China (48.1 nmol/L in Han children and 49.3 nmol/L in minority children, *P* = 0.587) [21]. Overweight and obesity are among the widely accepted risk factors for the serum vitamin D level. Overweight and obese children tend to live a sedentary life. Moreover, vitamin D through supplementation or cutaneous synthesis is volumetrically diluted and isolated by adipose tissues, possible resulting in the low serum vitamin D level in this population [22]. In addition, we also found that hypovitaminosis D was prevalent in older children, and infants had the highest level of mean serum 25(OH) D (33.25 ± 13.64 ng/ml). However, childhood vitamin D nutritional status deteriorated with advancing age, which was in line with Korean and Canadian studies [23, 24]. Generally, outdoor activities and vitamin D supplementation in children younger than 3 years old depend on parental support, whereas those in school children older than 6 years are limited or encouraged by schools. Thus, we divided children’s age into three groups and found that vitamin D intervention rate in group of age 0–3 years (66.89%) was higher than that of age 3–6 (42.04%) and above 6 years old (25.81%). The policy of physical examination by local communities is that regular physical examination free of charge is provided to children younger than 3 years old every 3–6 months and to those aged 3–6 years old every year, including screening for rickets and education on vitamin D supplementation. Nevertheless, routine physical examination is not performed in children older than 6 years old. Different health policy may be the main cause for high rate of vitamin D supplementation in younger children. Despite varying outdoor time and vitamin D interventions in each age group, an increase in outdoor time and vitamin D intervention present effectiveness in hypovitaminosis D prevention.

Latitude has a clear impact on the vitamin D status in children. However, we still lack of data for the vitamin D nutriture of children form different latitudes. Previous hospital-based cross-sectional studies indicated that 23.28% children in Huzhou (southeastern China, 30°2′–31°1′ N) [25] and 33.60% in Hangzhou (southeastern China, 29°1′–30°3′ N) [26] were found with hypovitaminosis D, whereas the prevalence increased to 65.91% in this study, which is consistent with early research focusing on children in high latitudes. Data from Hutterite communities (Canada, 49°2′–54°8′N) suggested that 76.00% of children suffered from hypovitaminosis D [27], indicating that hypovitaminosis D might be a common and serious problem in children in high latitudes. Skin is a key organ in vitamin D synthesis because 80–90% vitamin D that the human needs per day is produced in the skin from ultraviolet-B-activated 7-dehydrocholesterol [28]. Evidence from Spain (36°0′–43°2′ N) suggested that production of over 1000 IU/d vitamin D in spring and summer depends on regular sunlight exposure [29], making outdoor activity an optimal way of getting vitamin D. However, our results showed that still 17.75 and 21.63% of the enrolled children whose outdoor time was over 60 min/d had a deficient and insufficient vitamin D status, respectively. Moreover, excessive ultraviolet radiation exposure throughout childhood appears to be particularly harmful [30]. Children with sun protection by their parents are much less exposed to ultraviolet radiation [31]. Therefore, vitamin D supplementation might be taken into account to prevent hypovitaminosis D among children living in Heilongjiang.

Numerous programs or health policies for vitamin D intervention are available in almost all countries to prevent vitamin D deficiency or hypovitaminosis D in children [32–35]. In the guidelines aforementioned, some risk factors for vitamin D deficiency are recognized, including vegan diet, malabsorption syndromes, and reduced sunlight exposure; meanwhile, regular vitamin D supplementation and routine 25(OH) D testing are recommended for children. However, living at high latitude as an identified risk factor for vitamin D deficiency has not been explicitly mentioned for routine 25(OH) D testing in these guidelines. Despite of high incidence of hypovitaminosis D among children living in high latitudes was found in previous studies [36], few of them focused on related improvements. Approximately 30% of the total world population lives from 40° N to 60°N. Given the vitamin D status of children living at high latitudes, it is necessary to determine whether they need to be routinely screened for serum 25 (OH) D levels, followed by appropriate vitamin D intervention as required. In this study, we found that hypovitaminosis D was prevalent in children living at high latitude, regardless of seasons or outdoor time. The vast majority of children maintaining adequate vitamin D status took vitamin D supplements. Therefore, despite the recommendations by some guidelines that populations at high risk of vitamin D deficiency should be routinely screened, the results of our study support regular vitamin D intervention for local children regardless of taking the 25(OH) D test. However, more future research is needed to confirm whether all children living at high latitudes should be given routine vitamin D intervention without 25(OH) D test.

The pediatric health care system in China has been improved and consists of a proper examination interval and routine monitor network of serum 25(OH) D levels in children [25, 37]. Prescriptions were given by health professionals according to vitamin D status and the supplementary recommendation in the consensus of the Chinese Society of Osteoporosis and Bone Mineral Research. To the best of our knowledge, this was the first study to describe and evaluate the association of therapeutic intervention on prevention of hypovitaminosis D among children, and therapeutic intervention had a 2.21-, and 5.24-fold decreased risk of hypovitaminosis D compared with supplementation intervention and no supplementation, respectively. Besides, health professionals urge the parents to keep regular vitamin D supplementation [38]. Minkowitz et al. reported that better adherence of guardians with low serum vitamin D levels to physicians’ orders to remind their children of daily intake of vitamin supplements [39]. Although there were some unfavorable factors causing poor response to oral intervention [40], pediatricians could improve the oral doses according to the follow-up serum 25(OH) D test results until the doses reached optimum. Noticeably, however, compliance of children with follow-up blood collection varied. No vitamin D intervention due to guardians’ lack of awareness that might be the main cause [38], was like to expose their children to the increased risk of hypovitaminosis D and acute and chronic diseases including infectious diseases, autoimmune disorders and childhood dental caries [41]. Thus, although therapeutic intervention has shown a better effect in preventing hypovitaminosis D than voluntary supplementation, which prevention strategy to follow depends on certain factors, in particular the local health policy and compliance of guardians with blood collection.

This study had several limitations. First, the vitamin D doses in the therapeutic group remained inconsistent. Individualized vitamin D intervention were prescribed by doctors according to the medical conditions of healthy children and those with vitamin D deficiency. Second, outdoor time might be a confounder. However, duration and sun-exposed skin areas of each child was not investigated in this study. Third, < 10–20% vitamin D that human needed per day was from diet [42], which played an important role in the vitamin D status of children. However, such information was unavailable in our study. We assumed that children in the same region had similar dietary patterns. A prospective study is warranted to explore the role of diet in addition to other confounders. Fourth, as the subjects in this study were all recruited from the outpatient department instead of the general pediatric population, their parents might be more concerned about their children’s vitamin D status or health condition, and their health status may be better. Therefore, demographic characteristics of the participants in this study could not represent that of the whole population of children living in North China.

## Conclusions

In summary, a large number of blood tests in our study demonstrated low vitamin D status in local children. Supplementation and therapeutic vitamin D intervention were all promising strategies and prolonged outdoor time was negatively associated with pediatric vitamin D deficiency, the combination of which might be an optimal way to prevent hypovitaminosis D in children; nevertheless, guardian’s compliance might be considered for a practical prevention strategy. In addition, regardless of taking a 25(OH) D test, all children living at high latitudes might need routine vitamin D intervention.

## Supplementary Information

**Additional file 1: Supplement Figure**. Geographic characteristics of participants (city, number of subjects, %).

**Additional file 2: Supplement Table 1**. Logistic regression analysis of the association of intervention methods with hypovitaminosis D in each age group.

**Additional file 3: Supplement Table 2**. Logistic regression analysis of the association of outdoor time with hypovitaminosis D in each age group.

## Data Availability

The datasets used and/or analysed during the current study are available from the corresponding author on reasonable request.

## Abbreviations

25(OH)D: 25-Hydroxycholecalciferol
BMI: Body mass index
OR: Odds ratio
HPLC–MS/MS: High performance liquid chromatography tandem–mass spectrometry

## References

[1] Munns CF, Shaw N, Kiely M, Specker BL, Thacher TD, Ozono K, Michigami T, Tiosano D, Mughal MZ, Makitie O et al. Global consensus recommendations on prevention and management of nutritional rickets. J Clin Endocrinol Metab. 2016;101(2):394–415. 10.1210/jc.2015-2175. 10.1210/jc.2015-2175 PMC4880117 26745253

[2] Al-Jwadi RF, Jespersen E, Dalgard C, Bilenberg N, Christesen HT. S-25OHD is associated with hand grip strength and myopathy at 5 years in girls: an Odense child cohort study. J Clin Endocrinol Metab 2018; 103(7):2630–2639. 10.1210/jc.2018-00281. 10.1210/jc.2018-00281 29788436

[3] Smith EM, Tangpricha V. Vitamin D and anemia: insights into an emerging association. Curr Opin Endocrinol Diabetes Obes. 2015;22(6):432–438. 10.1097/med.0000000000000199. 10.1097/MED.0000000000000199 PMC4659411 26414080

[4] Zhang X, Ding F, Li H, Zhao W, Jing H, Yan Y, Chen Y (2016). Low serum levels of vitamins a, D, and E are associated with recurrent respiratory tract infections in children living in northern China: a case control study. PLoS One.

[5] Wang LL, Wang HY, Wen HK, Tao HQ, Zhao XW (2016). Vitamin D status among infants, children, and adolescents in southeastern China. J Zhejiang Univ Sci B.

[6] Peroni DG, Trambusti I, Di Cicco ME, Nuzzi G (2020). Vitamin D in pediatric health and disease. Pediatr Allergy Immunol.

[7] National Research Cooperation Group on rickets prevention and treatment (2015). Recommendation for prevention and treatment of rickets of vitamin D deficiency in childhood (Chinese). Chin J Child Health Care.

[8] Okazaki R, Ozono K, Fukumoto S, Inoue D, Yamauchi M, Minagawa M, Michigami T, Takeuchi Y, Matsumoto T, Sugimoto T. Assessment criteria for vitamin D deficiency/insufficiency in Japan: proposal by an expert panel supported by the research program of intractable diseases, Ministry of Health, Labour and Welfare, Japan, the Japanese Society for Bone and Mineral Research and the Japan Endocrine Society [opinion]. J Bone Miner Metab. 2017;35(1):1–5. 10.1007/s00774-016-0805-4. 10.1007/s00774-016-0805-4 27882481

[9] Schou AJ, Heuck C, Wolthers OD. Does vitamin D administered to children with asthma treated with inhaled glucocorticoids affect short-term growth or bone turnover? Pediatr Pulmonol. 2003;36(5):399–404. 10.1002/ppul.10379. 10.1002/ppul.10379 14520722

[10] Hradsky O, Soucek O, Maratova K, Matyskova J, Copova I, Zarubova K, Bronsky J, Sumnik Z. Supplementation with 2000 IU of cholecalciferol is associated with improvement of trabecular bone mineral density and muscle power in pediatric patients with IBD. Inflamm Bowel Dis. 2017;23(4):514–523. 10.1097/mib.0000000000001047. 10.1097/MIB.0000000000001047 28267045

[11] Uysalol M, Mutlu LC, Saracoglu GV, Karasu E, Guzel S, Kayaoglu S, Uzel N. Childhood asthma and vitamin D deficiency in Turkey: is there cause and effect relationship between them? Ital J Pediatr. 2013;39:78. 10.1186/1824-7288-39-78. 10.1186/1824-7288-39-78 PMC3892001 24330502

[12] Misra M, Pacaud D, Petryk A, Collett-Solberg PF, Kappy M. Vitamin D deficiency in children and its management: review of current knowledge and recommendations. Pediatrics. 2008;122(2):398–417. 10.1542/peds.2007-1894. 10.1542/peds.2007-1894 18676559

[13] Liu Y, Li X, Zhao A, Zheng W, Guo M, Xue Y, Wang P, Zhang Y. High prevalence of insufficient vitamin D intake and serum 25-hydroxyvitamin D in chinese school-age children: a cross-sectional study. Nutrients. 2018;10(7). 10.3390/nu10070822. 10.3390/nu10070822 PMC6073881 29949856

[14] Saggese G, Vierucci F, Prodam F, Cardinale F, Cetin I, Chiappini E, De’ Angelis GL, Massari M, Del Giudice EM, Del Giudice MM et al. Vitamin D in pediatric age: consensus of the Italian pediatric society and the Italian society of preventive and social pediatrics, jointly with the Italian federation of pediatricians. Ital J Pediatr. 2018;44(1):51. 10.1186/s13052-018-0488-7. 10.1186/s13052-018-0488-7 PMC5941617 29739471

[15] Tao RX, Meng DH, Li JJ, Tong SL, Hao JH, Huang K, Tao FB, Zhu P. Current recommended vitamin D prenatal supplementation and fetal growth: results from the China-Anhui birth cohort study. J Clin Endocrinol Metab. 2018;103(1):244–252. 10.1210/jc.2017-00850. 10.1210/jc.2017-00850 29096022

[16] O'Callaghan KM, Taghivand M, Zuchniak A, Onoyovwi A, Korsiak J, Leung M, Roth DE. Vitamin D in breastfed infants: systematic review of alternatives to daily supplementation. Adv Nutr. 2019;11(1):144–159. 10.1093/advances/nmz098. 10.1093/advances/nmz098 PMC7442322 31552417

[17] Marwaha RK, Mithal A, Bhari N, Sethuraman G, Gupta S, Shukla M, Narang A, Chadda A, Gupta N, Sreenivas V, Ganie MA. Supplementation with three different daily doses of vitamin D3 in healthy pre-pubertal school girls: a cluster randomized trial. Indian Pediatr. 2018;55(11):951–6. 10.1007/s13312-018-1416-z. 30587642

[18] Xia. W, Zhang. Z, Lin. H, Jin. X, Wei. Y, Fu. Q. Consensus recommendations on vitamin D and their analogs (Chinese). Chin J Osteoporos Bone Miner Res. 2018;11(1):1–19. 10.3969/j.issn.1674-2591.2018.01.001.

[19] Dobson R, Cock HR, Brex P, Giovannoni G. Vitamin D supplementation. Pract Neurol. 2018;18(1):35–42. 10.1136/practneurol-2017-001720. 10.1136/practneurol-2017-001720 28947637

[20] Stepman HC, Vanderroost A, Van Uytfanghe K, Thienpont LM. Candidate reference measurement procedures for serum 25-hydroxyvitamin D3 and 25-hydroxyvitamin D2 by using isotope-dilution liquid chromatography-tandem mass spectrometry. Clin Chem. 2011;57(3):441–448. 10.1373/clinchem.2010.152553. 10.1373/clinchem.2010.152553 21248072

[21] Hu Y, Chen J, Wang R, Li M, Yun C, Li W, Yang Y, Piao J, Yang X, Yang L: Vitamin D nutritional status and its related Factors for Chinese children and adolescents in 2010-2012. Nutrients. 2017;9(9). 10.3390/nu9091024. 10.3390/nu9091024 PMC5622784 28914773

[22] Savastano S, Barrea L, Savanelli MC, Nappi F, Di Somma C, Orio F, Colao A: Low vitamin D status and obesity: role of nutritionist. Rev Endocr Metab Disord. 2017;18(2):215–225. https://doi.org/10.1007/s11154-017-9410-7. 10.1007/s11154-017-9410-7 28229265

[23] Chung IH, Kim HJ, Chung S, Yoo EG. Vitamin D deficiency in Korean children: prevalence, risk factors, and the relationship with parathyroid hormone levels. Ann Pediatr Endocrinol Metab. 2014;19(2):86–90. 10.6065/apem.2014.19.2.86. 10.6065/apem.2014.19.2.86 PMC4114049 25077091

[24] Taseen K, Beaulieu G. Vitamin D levels and influencing predictors in refugee children in Sherbrooke (Quebec), Can Paediatr Child Health. 2017;22(6):307–311. 10.1093/pch/pxx092. 10.1093/pch/pxx092 PMC5804816 29479242

[25] Wang S, Shen G, Jiang S, Xu H, Li M, Wang Z, Zhang S, Yu Y. Nutrient status of vitamin D among chinese children. Nutrients. 2017;9(4). 10.3390/nu9040319. 10.3390/nu9040319 PMC5409658 28333101

[26] Zhu Z, Zhan J, Shao J, Chen W, Chen L, Li W, Ji C, Zhao Z: High prevalence of vitamin D deficiency among children aged 1 month to 16 years in Hangzhou, China BMC Public Health. 2012;12:126. 10.1186/1471-2458-12-126. 10.1186/1471-2458-12-126 PMC3312872 22330045

[27] Science M, Maguire JL, Russell ML, Smieja M, Walter SD, Loeb M. Prevalence and predictors of low serum 25-hydroxyvitamin D levels in rural Canadian children. Paediatr Child Health. 2017;22(3):125–129. 10.1093/pch/pxx007. 10.1093/pch/pxx007 PMC5804950 29479197

[28] Satue M, Ramis JM, Monjo M. UV-activated 7-dehydrocholesterol-coated titanium implants promote differentiation of human umbilical cord mesenchymal stem cells into osteoblasts. J Biomater Appl. 2016;30(6):770–779. 10.1177/0885328215582324. 10.1177/0885328215582324 25899927

[29] Serrano MA. Contribution of sun exposure to the vitamin D dose received by various groups of the Spanish population. Sci Total Environ. 2018;619-620:545–551. 10.1016/j.scitotenv.2017.11.036. 10.1016/j.scitotenv.2017.11.036 29156273

[30] Greinert R, de Vries E, Erdmann F, Espina C, Auvinen A, Kesminiene A, Schuz J. European code against Cancer 4th edition: ultraviolet radiation and cancer. Cancer Epidemiol. 2015;39 Suppl 1:S75–S83. 10.1016/j.canep.2014.12.014. 10.1016/j.canep.2014.12.014 26096748

[31] Vierucci F, Del Pistoia M, Fanos M, Gori M, Carlone G, Erba P, Massimetti G, Federico G, Saggese G. Vitamin D status and predictors of hypovitaminosis D in Italian children and adolescents: a cross-sectional study. Eur J Pediatr. 2013;172(12):1607–1617. 10.1007/s00431-013-2119-z. 10.1007/s00431-013-2119-z 23959324

[32] Vitamin D Supplementation: recommendations for Canadian mothers and infants. Paediatr Child Health. 2007;12(7):583–598. 10.1093/pch/12.7.583. PMC2528771 19030432

[33] Braegger C, Campoy C, Colomb V, Decsi T, Domellof M, Fewtrell M, Hojsak I, Mihatsch W, Molgaard C, Shamir R et al. Vitamin D in the healthy European paediatric population. J Pediatr Gastroenterol Nutr. 2013;56(6):692–701. 10.1097/mpg.0b013e31828f3c05. 10.1097/MPG.0b013e31828f3c05 23708639

[34] Holick MF, Binkley NC, Bischoff-Ferrari HA, Gordon CM, Hanley DA, Heaney RP, Murad MH, Weaver CM. Evaluation, treatment, and prevention of vitamin D deficiency: an Endocrine Society clinical practice guideline. J Clin Endocrinol Metab. 2011;96(7):1911–1930. 10.1210/jc.2011-0385. 10.1210/jc.2011-0385 21646368

[35] Mo M, Wang S, Chen Z, Muyiduli X, Shen Y, Shao B, Li M, Chen D, Yu Y. A systematic review and meta-analysis of the response of serum 25-hydroxyvitamin D concentration to vitamin D supplementation from RCTs from around the globe. Eur J Clin Nutr. 2019;73(6):816–834. 10.1038/s41430-019-0417-x. 10.1038/s41430-019-0417-x 30872787

[36] Karin Z, Gilic B, Supe Domic D, Sarac Z, Ercegovic K, Zenic N, Uljevic O, Peric M, Markic J. Vitamin D status and analysis of specific correlates in preschool children: a cross-sectional study in southern Croatia. Int J Environ Res Public Health. 2018;15(11):2053. 10.3390/ijerph15112503. 10.3390/ijerph15112503 PMC6266977 30413103

[37] Liang Y, Ren HY, Zuo PX. Associations between maternal nutrition knowledge, attitude, and practice and 25-hydroxyvitamin D levels and rickets in children in Xinjiang province, People's Republic of China. Asia Pac J Public Health. 2018;30(4):378–386. 10.1177/1010539518768034. 10.1177/1010539518768034 29652179

[38] Day RE, Krishnarao R, Sahota P, Christian MS. We still don't know that our children need vitamin D daily: a study of parents' understanding of vitamin D requirements in children aged 0-2 years. BMC Public Health. 2019;19(1):1119. 10.1186/s12889-019-7340-x. 10.1186/s12889-019-7340-x PMC6694627 31416429

[39] Minkowitz B, Nadel L, McDermott M, Cherna Z, Ristic J, Chiu S. Obtaining vitamin D levels in children with fractures improves supplementation compliance. J Pediatr Orthop. 2019;39(6):e436-e440. 10.1097/bpo.0000000000001363. 10.1097/BPO.0000000000001363 30855552

[40] Petersen RA, Larsen LH, Damsgaard CT, Sorensen LB, Hjorth MF, Andersen R, Tetens I, Krarup H, Ritz C, Astrup A et al. Common genetic variants are associated with lower serum 25-hydroxyvitamin D concentrations across the year among children at northern latitudes. Br J Nutr. 2017;117(6):829–838. 10.1017/s0007114517000538. 10.1017/S0007114517000538 28382877

[41] Holick MF. The vitamin D deficiency pandemic: approaches for diagnosis, treatment and prevention. Rev Endocr Metab Disord. 2017;18(2):153–165. 10.1007/s11154-017-9424-1. 10.1007/s11154-017-9424-1 28516265

[42] Reichrath J, Saternus R, Vogt T. Challenge and perspective: the relevance of ultraviolet (UV) radiation and the vitamin D endocrine system (VDES) for psoriasis and other inflammatory skin diseases. Photochem Photobiol Sci. 2017;16(3):433–444. 10.1039/c6pp00280c. 10.1039/c6pp00280c 28054069

