# Supplementary figures and images for: Association of serum 25-Hydroxyvitamin D with Vitamin D intervention and outdoor activity among children in North China: an observational study
Source: BMC Pediatr. 2020 Dec 2;20:542. doi: 10.1186/s12887-020-02435-9 (PMC7709348; doi:10.1186/s12887-020-02435-9)

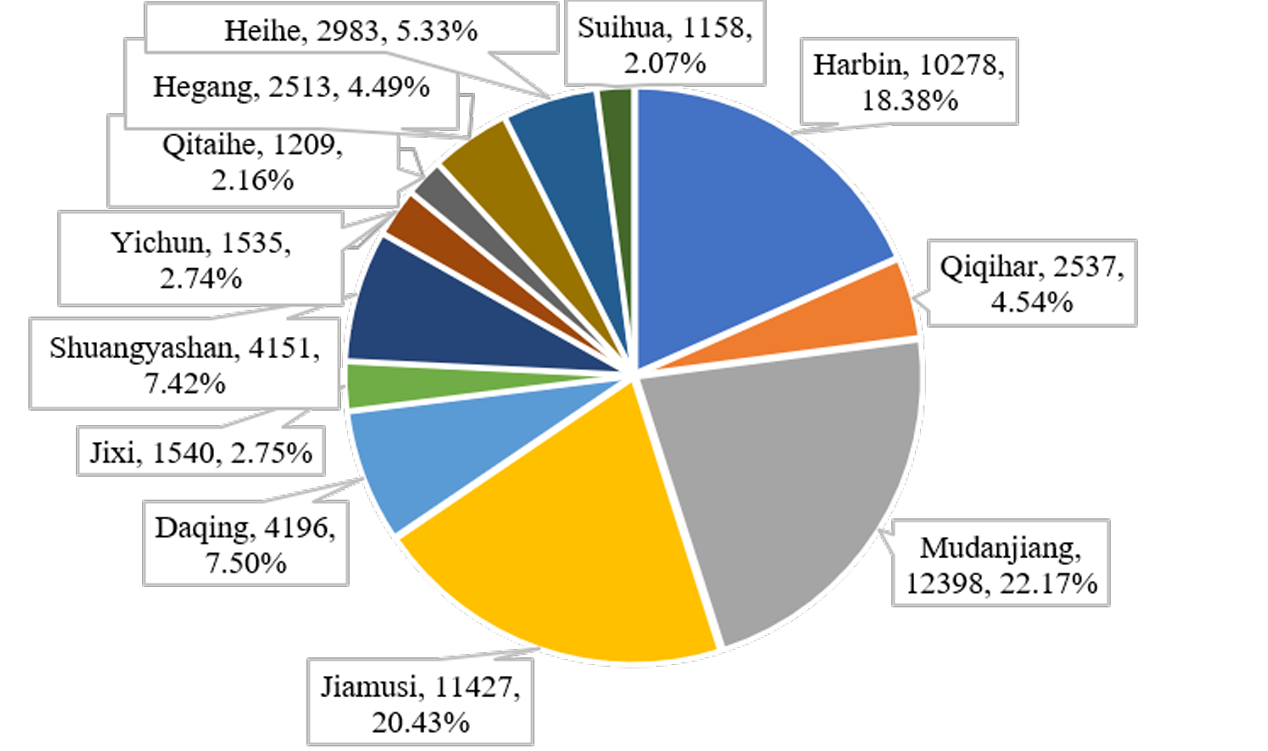


Supplement. Figure. Geographic characteristics of participants (city, number of subjects, %).

Supplement: Supplementary file 1 — Additional file 1: Supplement Figure. Geographic characteristics of participants (city, number of subjects, %). [file 12887_2020_2435_MOESM1_ESM.docx]
