# Supplementary material for: Association of serum 25-Hydroxyvitamin D with Vitamin D intervention and outdoor activity among children in North China: an observational study
Source: BMC Pediatr. 2020 Dec 2;20:542. doi: 10.1186/s12887-020-02435-9 (PMC7709348; doi:10.1186/s12887-020-02435-9)
Supplement: Supplementary file 2 — Additional file 2: Supplement Table 1. Logistic regression analysis of the association of intervention methods with hypovitaminosis D in each age group. [file 12887_2020_2435_MOESM2_ESM.docx]

**Supplement. Table.1 Logistic regression analysis of the association of intervention methods with hypovitaminosis D in each age group**

| Intervention methods | n (%) | Model 1 | Model 2 | Model 3 |
| --- | --- | --- | --- | --- |
| 0< age <3 |  |  |  |  |
| No intervention | 8891 (33.11) | 1 | 1 | 1 |
| Supplementation intervention | 13002 (48.42) | 0.359 (0.339, 0.380) | 0.359 (0.340, 0.380) | 0.359 (0.340, 0.380) |
| Therapeutic intervention | 4961 (18.47) | 0.188 (0.174, 0.203) | 0.188 (0.174, 0.203) | 0.181 (0.167, 0.196) |
| 3≤ age <6 |  |  |  |  |
| No intervention | 7606 (57.96) | 1 | 1 | 1 |
| Supplementation intervention | 3237 (24.66) | 0.561 (0.505, 0.624) | 0.561 (0.505, 0.624) | 0.460 (0.412, 0.514) |
| Therapeutic intervention | 2281 (17.38) | 0.244 (0.220, 0.272) | 0.244 (0.220, 0.272) | 0.208 (0.186, 0.232) |
| 6≤ age <18 |  |  |  |  |
| No intervention | 11831 (74.19) | 1 | 1 | 1 |
| Supplementation intervention | 2144 (13.44) | 0.773 (0.641, 0.932) | 0.775 (0.643, 0.934) | 0.758 (0.629, 0.914) |
| Therapeutic intervention | 1972 (12.37) | 0.150 (0.132, 0.170) | 0.149 (0.132, 0.170) | 0.150 (0.132, 0.171) |

Associations were examined using multivariable logistic regression. Model 1: adjusted for sex. Model 2: adjusted for BMI for age on the basis of model 1. Model 3: adjusted for season and outdoor time on the basis of model 2.
