# Supplementary material for: Association of serum 25-Hydroxyvitamin D with Vitamin D intervention and outdoor activity among children in North China: an observational study
Source: BMC Pediatr. 2020 Dec 2;20:542. doi: 10.1186/s12887-020-02435-9 (PMC7709348; doi:10.1186/s12887-020-02435-9)
Supplement: Supplementary file 3 — Additional file 3: Supplement Table 2. Logistic regression analysis of the association of outdoor time with hypovitaminosis D in each age group. [file 12887_2020_2435_MOESM3_ESM.docx]

**Supplement. Table.2 Logistic regression analysis of the association of outdoor time with hypovitaminosis D in each age group**

| Outdoor time | n (%) | Model 1 | Model 2 | Model 3 |
| --- | --- | --- | --- | --- |
| 0< age <3 |  |  |  |  |
| <30 | 7563 (28.16) | 1 | 1 | 1 |
| 30–60 | 9560 (35.60) | 0.684 (0.643, 0.727) | 0.685(0.645, 0.728) | 0.718 (0.673, 0.765) |
| >60 | 9731 (36.24) | 0.605 (0.569, 0.643) | 0.606(0.570, 0.644) | 0.567 (0.532, 0.605) |
| 3≤ age <6 |  |  |  |  |
| <30 | 5138 (39.15) | 1 | 1 | 1 |
| 30–60 | 3746 (28.54) | 0.690 (0.616, 0.774) | 0.688 (0.614, 0.772) | 0.747 (0.664, 0.840) |
| >60 | 4240 (32.31) | 0.382 (0.344, 0.423) | 0.379 (0.342, 0.420) | 0.302 (0.271, 0.338) |
| 6≤ age <18 |  |  |  |  |
| <30 | 7228 (45.33) | 1 | 1 | 1 |
| 30–60 | 4561 (28.60) | 0.882 (0.738, 0.929) | 0.864 (0.752, 0.915) | 0.821 (0.764, 0.902) |
| >60 | 4158 (26.07) | 0.663 (0.557, 0.721) | 0.634 (0.557, 0.721) | 0.626 (0.547, 0.715) |

Associations were examined using multivariable logistic regression. Model 1: adjusted for sex. Model 2: adjusted for BMI for age on the basis of model 1. Model 3: adjusted for season and intervention methods on the basis of model 2.
